# Comparison of 3DCRT and IMRT out-of-field doses in pediatric patients using Monte Carlo simulations with treatment planning system calculations and measurements

**DOI:** 10.3389/fonc.2022.879167

**Published:** 2022-08-05

**Authors:** Ana Cravo Sá, Andreia Barateiro, Bryan P. Bednarz, Pedro Almeida, Pedro Vaz, Tiago Madaleno

**Affiliations:** ^1^ Radiation Protection and Safety Group, Centro de Ciências e Tecnologias Nucleares (C2TN), Bobadela, Portugal; ^2^ Diagnostic, Therapeutic and Public Health Sciences Department, Escola Superior de Tecnologia da Saúde de Lisboa (ESTeSL), Lisbon, Portugal; ^3^ Instituto de Biofísica e Engenharia Biomédica, Faculdade de Ciências, Universidade de Lisboa, Lisbon, Portugal; ^4^ Radiotherapy Department, Portuguese Institute of Oncology Francisco Gentil, Lisbon, Portugal; ^5^ Department of Medical Physics, Wisconsin Institutes for Medical Research, University of Wisconsin Hospital and Clinics, Madison, WI, United States

**Keywords:** radiotherapy planning, out-of-field dose, pediatric tumors, Monte Carlo simulations, computational voxel phantoms, IMRT, 3DCRT

## Abstract

3DCRT and IMRT out-of-field doses in pediatric patients were compared using Monte Carlo simulations with treatment planning system calculations and measurements.

**Purpose:**

Out-of-field doses are given to healthy tissues, which may allow the development of second tumors. The use of IMRT in pediatric patients has been discussed, as it leads to a “bath” of low doses to large volumes of out-of-field organs and tissues. This study aims to compare out-of-field doses in pediatric patients comparing IMRT and 3DCRT techniques using measurements, Monte Carlo (MC) simulations, and treatment planning system (TPS) calculations.

**Materials and methods:**

A total dose of 54 Gy was prescribed to a PTV in the brain of a pediatric anthropomorphic phantom, for both techniques. To assess the out-of-field organ doses for both techniques, two treatment plans were performed with the 3DCRT and IMRT techniques in TPS. Measurements were carried out in a LINAC using a pediatric anthropomorphic phantom and thermoluminescent dosimeters to recreate the treatment plans, previously performed in the TPS. A computational model of a LINAC, the associated multileaf collimators, and a voxelized pediatric phantom implemented in the Monte Carlo N-Particle 6.1 computer program were also used to perform MC simulations of the out-of-field organ doses, for both techniques.

**Results:**

The results obtained by measurements and MC simulations indicate a significant increase in dose using the IMRT technique when compared to the 3DCRT technique. More specifically, measurements show higher doses with IMRT, namely, in right eye (13,041 vs. 593 mGy), left eye (6,525 vs. 475 mGy), thyroid (79 vs. 70 mGy), right lung (37 vs. 28 mGy), left lung (27 vs. 20 mGy), and heart (31 vs. 25 mGy). The obtained results indicate that out-of-field doses can be seriously underestimated by TPS.

**Discussion:**

This study presents, for the first time, out-of-field dose measurements in a realistic scenario and calculations for IMRT, centered on a voxelized pediatric phantom and an MC model of a medical LINAC, including MLC with log file-based simulations. The results pinpoint significant discrepancies in out-of-field doses for the two techniques and are a cause of concern because TPS calculations cannot accurately predict such doses. The obtained doses may presumably increase the risk of development of second tumors.

## Introduction

In photon radiotherapy, out-of-field doses are mainly caused by radiation scattered in the collimators, radiation leakage from the linear accelerator head, and radiation scattered inside the patient’s body (1–10). Out-of-field doses are non-target doses that are outside of the planning target volume (PTV) and also outside the primary field edge (4). These doses are often disregarded in radiotherapy treatment planning, because they are considered “low doses” (4). The purpose of radiotherapy is to irradiate a tumor volume with high doses. Doses below 5% of the total dose prescribed or doses below 3 Gy are considered low doses (4) which are important because they can increase the probability of development of a second cancer (2, 11). The development of a second cancer probability increases when high doses are outside the treatment field, even in the tissues closest to the PTV; however, low doses further from the PTV cannot be ignored. This is particularly important in pediatric patients (2, 12), because children are considered to be a factor of 10 times more sensitive to radiation (12), when compared to adults. The higher radiosensitivity found in children can be attributed to several factors, such as higher cell proliferation in pediatric ages, higher susceptibility of normal tissues to the mutagenic effects of ionizing radiation in children, and genetic susceptibility related to some primary tumors (13). In addition, radiation-induced tumors may develop in organs contained within the treatment fields, e.g., in high-dose regions, or even in organs distant from the treatment fields exposed to lower radiation doses (12).

According to the National Council on Radiation Protection and Measurements (NCRP) report 116 (14), the intestine, lung, and stomach are the most common sites for the development of second tumors after exposure to radiation (12, 14). However, the thyroid is also known to have a low tolerance to radiation, especially in children (12, 15). An increased incidence of thyroid cancer has been reported after exposure to an average dose of 0.05 Gy in children and young adults (12).

The increasing use of intensity-modulated radiotherapy (IMRT) techniques, such as volumetric-modulated arc therapy (VMAT), will lead to a higher risk of developing second tumors, given the administration of low doses to large volumes outside the treatment fields (10). Using IMRT, the total number of monitor units (MU) is usually higher for the treatment of similar cases when compared to 3D conformal radiotherapy (3DCRT) (7, 16, 17). Furthermore, the importance of knowing the variation of the doses as a function of the distance from the field edge was highlighted in a very recent study (18), where the authors provide a model for determining the out-of-field doses as a function of the distance from the field edge. In addition, the need to study and understand how the treatment planning system (TPS) accuracy impacts out-of-field doses in pediatric radiotherapy was emphasized in (19).

In our previous study (20), out-of-field doses for the 3DCRT technique were evaluated by measurements, MC simulations, and TPS calculations. To assess the out-of-field doses with the 3DCRT technique, we initially validated an MC model of a Varian 2100 linear accelerator and then we performed dose measurements with thermoluminescent dosimeters (TLDs) on a pediatric anthropomorphic phantom, based on the treatment planning performed in the TPS. In the present study, these previously obtained results only provide a comparison with the new results obtained for the IMRT technique.

Ruben et al., in 2011, compared 3DCRT with IMRT, concluding that the out-of-field doses with IMRT increases for i) smaller field dimensions, ii) higher MU, and iii) higher distance from the field edge (16). The same authors concluded that IMRT yields a higher total dose of scattered radiation in the patient than 3DCRT (16). Additionally, in another study, other authors found that the IMRT increases the dose inside the patient’s body, when compared with 3DCRT, and may presumably double the incidence of solid tumors in long-term survivors (3).

The aim of this study was to compare out-of-field doses in 3DCRT and IMRT treatments of pediatric patients, using the MC model of a linear accelerator (LINAC) head and associated multileaf collimators (MLCs), coupled to a computational pediatric voxel phantom developed and validated (20) from a physical phantom. To the best of our knowledge, no comparison between out-of-field doses in 3DCRT and IMRT using thermoluminescent dosimeter (TLD) measurements, MC simulations, and TPS calculations was yet undertaken prior to our study.

Our study accurately mimics a treatment with the IMRT technique applied to a pediatric case. To reproduce the dynamic movement of each leaf of the MLC during irradiation, we implemented an MC model of a LINAC. Several cutting-edge features of this work must be emphasized: i) a computational pediatric anthropomorphic phantom created from the original computed tomography (CT) images combined with an MC model of a LINAC head and MLC was used to calculate the organ doses by MC log file-based simulations for the IMRT technique; ii) the CT images of the pediatric phantom were used to calculate the organ doses with the treatment planning system (TPS) for the IMRT technique; and iii) TLD measurements in the physical pediatric anthropomorphic phantom were used to obtain the organ doses with the IMRT technique, creating a realistic scenario for treatment delivery. The combination of the listed features and methods allowed for an accurate comparison between 3DCRT and IMRT out-of-field doses using MC simulations, TPS calculations, and TLD measurements performed in a clinical environment, highlighting the innovation of this study, compared to those described in the literature.

## Materials and methods

### Treatment planning

An Atom^®^ 5-year-old physical pediatric phantom from CIRS, named George, with 110 cm of height and 19 kg of weight was the anthropomorphic pediatric used in this work. Considering that the phantom lacks a tumor volume, an elliptically shaped PTV was defined with 9.8 cm (3) in the right hemisphere of the brain. The volume and shape of the tumor were based on the analysis of 47 pediatric clinical cases, aged between 4 and 7 years. The organs at risk (OARs) segmented were the lungs, thyroid, heart, C-spine, and eyes. These OARs were chosen essentially for two reasons: i) low dose-induced biological effects to the OARs could affect function/growth; ii) OARs are well defined in the anthropomorphic phantom used in this study. In the TPS, the distance between the PTV and the different OARs evaluated was calculated by selecting the geometric center of each volume and then obtaining the distance between each of them.

**Figure 1:**
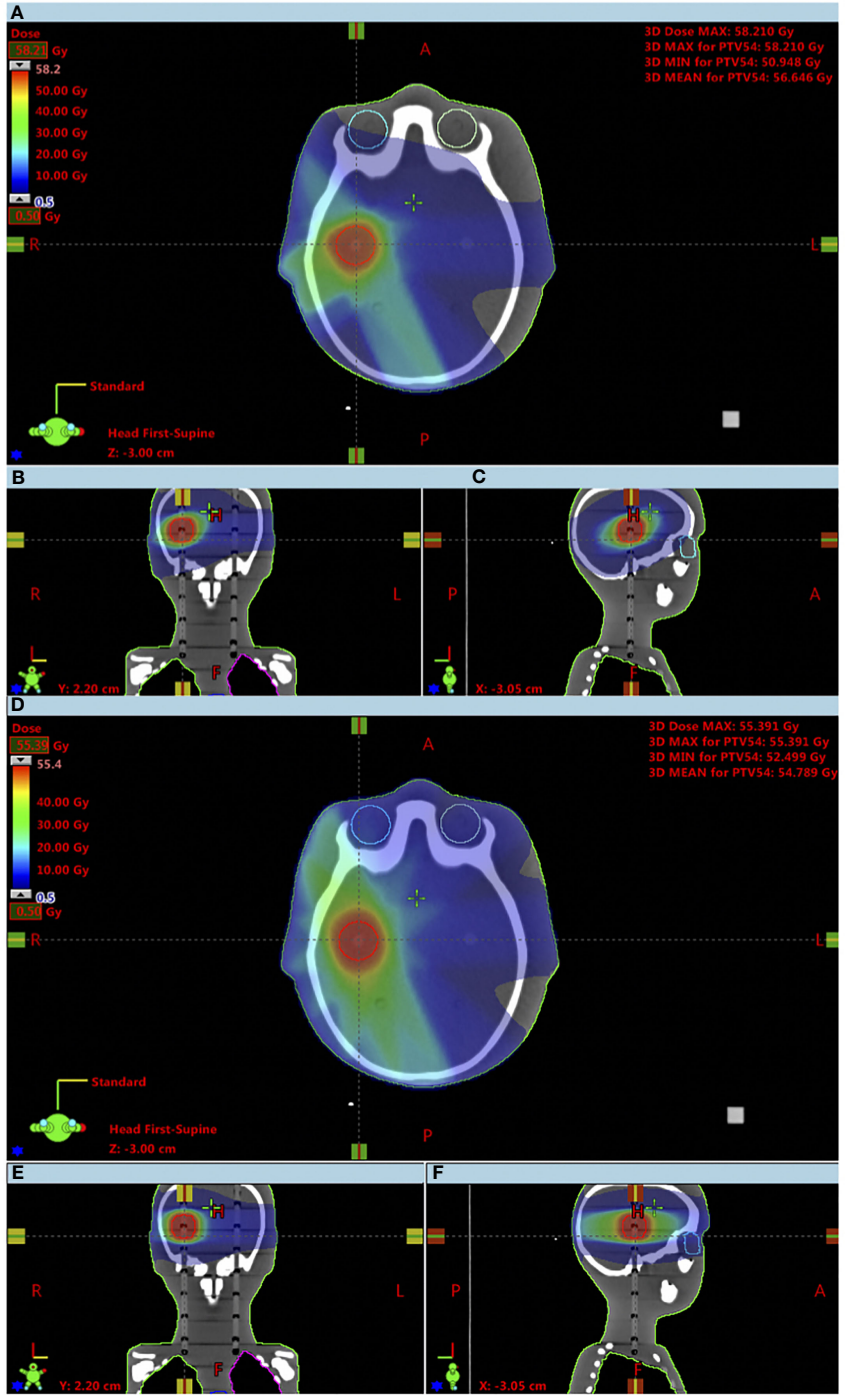
Comparison of obtained dose distributions between the 3DCRT and IMRT techniques up to a minimum dose of 500 mGy. **(A)** 3DCRT axial view; **(B)** 3DCRT coronal view; **(C)** 3DCRT sagittal view; **(D)** IMRT axial view; **(E)** IMRT coronal view; **(F)** IMRT sagittal view.

A treatment planning was performed using the 3DCRT and IMRT techniques, for a 6-MV photon beam and with a total prescribed dose to the PTV of 54 Gy, with a dose per fraction of 1.8 Gy in both cases, as shown in **Figure 1**. The treatment plans were executed by the Eclipse TPS from Varian (Varian Medical Systems, Palo Alto, CA) Version 13.0 and using version 13.6.23 of the analytical anisotropic algorithm (AAA) dose calculation algorithm. The AAA was used to calculate organ mean doses. The 3DCRT treatment plan was created using six non-coplanar brain fields. The treatment field details are displayed in **Table 1**. For the OARs of this study, the QUANTEC tables were used (21–25), for both techniques. The IMRT treatment plan was created using seven coplanar brain fields. The treatment field details are displayed in **Table 2**. For MLC, the dynamic mode was used, and the progressive resolution optimizer performed the optimization of the dose calculation. Considering a brain irradiation and the previously segmented volumes, the dose objectives were defined for the eyes, since the eyes are the volumes of risk closest to PTV. In addition, in clinical environment, only the eyes would be considered as OARs, as shown in **Table 3**. The phantom was irradiated in the same LINAC under the conditions previously described, for the two techniques.

**Table 1:** 3DCRT planning dose parameters.

Field ID	Gantry (deg)	Collimator (deg)	Couch (deg)	Field X (cm)	X1 (cm)	X2 (cm)	Field Y (cm)	Y1 (cm)	Y2 (cm)	Field weight	SSD (cm)	MU
**1**	320.0	0.0	0.0	3.2	+1.6	+1.6	3.2	+1.6	+1.6	0.70	95.1	32
**2**	270.0	0.0	0.0	3.2	+1.6	+1.6	3.2	+1.6	+1.6	0.70	96.5	30
**3**	235.0	0.0	15.0	3.4	+1.7	+1.7	3.4	+1.7	+1.7	0.70	95.9	31
**4**	40.0	0.0	50.0	3.4	+1.7	+1.7	3.4	+1.7	+1.7	1.10	90.1	63
**5**	155.0	0.0	345.0	3.4	+1.7	+1.7	3.4	+1.7	+1.7	1.00	91.5	53
**6**	60.0	309.0	30.0	3.4	+1.7	+1.7	3.5	+1.7	+1.8	1.00	89.3	60

**Table 2:** IMRT planning dose parameters.

Field ID	Gantry (deg)	Collimator (deg)	Couch (deg)	Field X (cm)	X1 (cm)	X2 (cm)	Field Y (cm)	Y1 (cm)	Y2 (cm)	Field weight	SSD (cm)	MU
**1**	340.0	0.0	0.0	5.0	+2.5	+2.5	3.6	+1.8	+1.8	1.0	93.5	51.0
**2**	314.0	0.0	0.0	5.0	+2.5	+2.5	3.6	+1.8	+1.8	1.0	95.4	47.0
**3**	266.0	0.0	0.0	5.0	+2.5	+2.5	3.6	+1.8	+1.8	1.0	96.5	43.0
**4**	228.0	0.0	0.0	5.0	+2.5	+2.5	3.6	+1.8	+1.8	1.0	95.8	43.0
**5**	197.0	0.0	0.0	5.0	+2.5	+2.5	3.6	+1.8	+1.8	1.0	94.2	47.0
**6**	173.0	0.0	0.0	5.0	+2.5	+2.5	3.6	+1.8	+1.8	1.0	92.7	50.0
**7**	154.0	0.0	0.0	5.0	+2.5	+2.5	3.6	+1.8	+1.8	1.0	91.5	53.0

**Table 3:** Optimization objectives for inverse planning.

Structure	Limit	Volume (%)	Dose (Gy)	Priority
**Left eye**	Upper	0.0	5.0	60
	Upper	5.0	3.0	60
**Right eye**	Upper	0.0	8.0	70
	Upper	5.0	6.0	70
**PTV**	Upper	0.0	55.1	220
	Lower	99.0	54.3	280
	Lower	100.0	54.00	280

Limit: constraints expressed as lower or upper dose limits for organs. Priority: priority in the optimization goal.

### TLD measurements using a pediatric phantom

In this study, Harshaw Ext-Rad (LiF : Mg,Cu,P) TLDs were placed in the eyes, lungs, heart, thyroid, and C-spine of the pediatric phantom. A total of 76 dosimeters, divided into two groups of 38 dosimeters, were used. The first group of dosimeters was irradiated with the 3DCRT technique, and the second group of dosimeters was irradiated with the IMRT technique. Each dosimeter has a sensitive diameter of 0.5 cm, a length of 5.1 cm, a height of 1.34 cm, and a thickness of 0.1 cm. For each group, one dosimeter was placed in the right eye, one for the left eye, two for the c-spine, four for the thyroid, two for the heart, 12 for the right lung, and 16 for the left lung.

The TLDs were previously calibrated using air kerma with a Cs-137 source in a reference metrology laboratory. The day before irradiation, the TLDs were reset. The day after irradiation, readings were performed using a Harshaw 6600 reader with a previously defined temperature and time profile in order to avoid contributions from non-dosimetric peaks (26), and a preheating was performed. Transit dosimeters were used, but since their value was negligible, background subtraction was not performed.

The kerma in air (*K_air_*) was calculated using the following equation (27):


(1) $$K_{air}\;=\;\frac{RD\;\times \;Ecc}{RCF}\;\times \;f\left(Q\right)\times \;f\left(fad\right)\times \;f\left(E\right)\times \;f\left(\alpha \right)$$

For each TLD, the raw data (*RD*) is multiplied by the element correction coefficient (*Ecc*), the correction factors of reader stability (*f*(*Q*)), fading effect (*f*(*fad*)), energy dependency (*f*(*E*)), and angular dependency (*f*(*α*)) and divided by the reader calibration factor (*RCF*).

The interval between the reset and the readout was negligible, and for this reason the correction due to the fading effect was not considered. The angular dependence correction factor was considered equal to 1, since TLDs have no angular dependence for the energy threshold (28).

The final dose value assessed at each position of the TLDs in the pediatric phantom was obtained based on the following equation (27), assuming that the electronic equilibrium condition is observed:


(2) $$D_{tissue}\;=\;K_{air}\;\times \;\frac{{\left(\mu_{en}/\rho \right)}_{tissue}}{{\left(\mu_{en}/\rho \right)}_{air}}$$

 where *K_air_* was previously defined, (*μ_en_*/*ρ*)*_tissue_* is the mass energy-absorption coefficient for each tissue, and (*μ_en_*/*ρ*)*_air_* is the mass energy-absorption coefficient for air at an average energy of the photon spectrum of 6 MeV. The mass energy-absorption coefficients for air and tissues were obtained through a web-based National Institute of Standards and Technology (NIST) (29), as is seen in **Table 4**.

**Table 4:** Organ characteristics assessed in phantom.

Organ	μ_en_/ρ (cm^2^/g) @ 6 MeV	Nr. of phantom sections	Nr. of TLDs
**Right eye**	0.0179	1	1
**Left eye**	0.0179	1	1
**C-spine**	0.0179	2	2
**Thyroid**	0.0194	2	4
**Heart**	0.0179	2	2
**Right lung**	0.0179	5	12
**Left lung**	0.0179	5	16

To calculate the final dose for each tissue, whenever there is more than one TLD per organ, the average of the dose readings of the TLDs for a given organ was performed.

The final relative uncertainty of the measurements was ≈16% (k = 1), calculated using the law of propagation of uncertainties, as the square root of the sum of the uncertainties squared (30) from the following contributions: (a) element correction coefficient (3.0%), (b) correction factors of reader stability (3.8%), (c) reader calibration factor (15.0%), and (d) energy dependency (1.4%) (26). The uncertainty value associated with each parameter was calculated using the maximum and minimum values of the variation interval obtained in each one of them during the time of uncertainty assessment.

Different probability distribution functions were used, depending on the expected distribution of the results. For a), the contribution of the element correction coefficient to the final uncertainty was obtained considering the stability of this factor along time. The stability of this factor was evaluated for 10 irradiation cycles, and the difference, for each detector, between the value obtained in each cycle and the previously dosimeter efficiency determined value was evaluated, assuming that the results present a Gaussian distribution; for b), the contribution from correction factors of reader stability was taken into consideration as well as the range of values obtained in quality control dosimeters during the period of 1 year, assuming a normal distribution of the results obtained; for c), the uncertainty was associated with the reader calibration factor results from the experimental history of the reader calibration factor over time and the uncertainty of the irradiance was reported by the Ionizing Radiation Metrology Laboratory of the Instituto Superior Técnico – Lisbon University; and for d), the energy dependence, it was considered that a dosimeter in normal routine conditions may be exposed to different radiation beams, and a rectangular distribution (α/√3) was assumed since all the energies studied for the effect have equal probability of occurrence.

The energy dependence of TLDs is often assumed to be small across the range of photon energies of relevance for this study, since TLDs are nearly energy independent for treatment energies (31). Although there is a dependence on energy at greater distances, energy dependence was considered to be low, because we evaluated doses up to a distance of 20 cm. Its accurate assessment is difficult due to the sizable uncertainties on the spectra of the photon field and its effective energy in organs located outside the main radiation field in external radiotherapy. Detailed information about the energy dependence of TLDs and other dosimeters can be found in (32).

The reader calibration factor considers the results of the last years for the calibration factors as well as the uncertainty mentioned by the metrology laboratory.

### MCNP6 Monte Carlo out-of-field dose simulations

For the IMRT technique, the MC simulations of the out-of-field doses in the organs were performed using the state-of-the-art computational program Monte Carlo N-Particle, version 6 (MCNP6) (33), using the developed pediatric voxel phantom developed in a previous study (20) and the implemented and validated LINAC head model (20). The pediatric voxel phantom was created from the CT images of a 5-year-old physical ATOM phantom™. The ImageJ software™ was used to build the phantom, considering structures such as the heart, lungs, eyes, soft tissues, thyroid, PTV, brain, whole body, bones, skin, spinal canal, and c-spine. In the end, a pediatric computational phantom was obtained with about 47 million voxels, each with a dimension of x = 0.09766 cm, y = 0.09766 cm, and z = 0.3 cm.

The IMRT MC simulations were performed in parallel processing mode with 10 × 10 (9) photons produced in the target. To model the 6-MV energy photon beam, a fine-tuning process was performed, in order to adjust the parameters previously described in other studies (34–39), such as the primary electron energy and the full width half maximum (FWHM) of the Gaussian beam intensity distribution. MC simulations were performed for different values of the primary electron energy and different values of FWHM. By comparing the measured and calculated depth dose profiles and beam profiles (40–43), the electron beam’s energy of 6.2 MeV and the FWHM of 1.2 mm were selected. The source definition card (SDEF) was used to specify a single-beam source of photons from the target (option available in MCNP), as a source distribution function traveling along the z-axis. The electron and photon energy cutoffs were set to 0.1 and 0.01 MeV, respectively. The data libraries available from ENDF/B-VII were used for particle transport simulation. As for the implemented variance reduction techniques, “Russian roulette” together with splitting was used for all MC simulations. The tally *F8 was used for scoring the results, and a statistical relative uncertainty of the computational results of less than 5% for 1σ was obtained.

For the 3DCRT technique, MC simulations were made for the six fields with the MLC positions described in **Table 2**. For IMRT, the positions of the MLCs were obtained through the MLC log file of each field. These log files were extracted from the Varian TPS Eclipse system, and each log file contains information for about 100 MLC positions for each field. There are about 700 positions of the MLC for the seven treatment fields of the IMRT plan. In order to minimize the computational effort, 20 MLC positions were selected for each field, totaling 140 simulations.

As in 3DCRT (**Table 2**), the movement of the leaves in IMRT appears only between the pair 27 and 34. Based on the log files and for each IMRT field, the indexes 5, 10, 15, 20, 25, 30, 35, 40, 45, 50, 55, 60, 65, 70, 75, 80, 85, 90, 95, and 100 were selected. In order to change the position of the leaves in MCNP6, it was necessary to use the cell coordinate transformation (*TRCL) card together with the surface coordinate transformation (*TR) card, to create the rotation of the leaves and, therefore, obtain different MLC positions based on the different secondary collimators (33).

In addition, as discussed by Frank Verhaegen (44), for each beam energy created by a LINAC a conversion factor (CF) can be obtained. Considering that the results in the MCNP6 are normalized per source particle, we used the previously calculated CF to achieve the absolute dose in mGy (20). These values were measured and calculated considering the SSD of 100 cm between the source and the water phantom in the central axis of the beam, under the reference conditions, i.e., 10 × 10 cm2 field size with the MLC retracted. It is possible to use the same CF, since the MC model is the same and only the setup of the simulations varies, because the position of the MLCs in each simulation varies. All values obtained with the tally *F8 in MCNP6 for organs were multiplied by the CF.

In order to compare the three different approaches, it was defined that the relative differences are calculated as,


(3), $$Relative\;Differences=\;\frac{\left|Calculations-Measurements\right|}{Measurements}\;\times \;100$$

where *Calculations* is related both to MCNP6 and TPS calculations.

## Results

All results are given per prescribed dose of 54 Gy.

**Figure 2:**
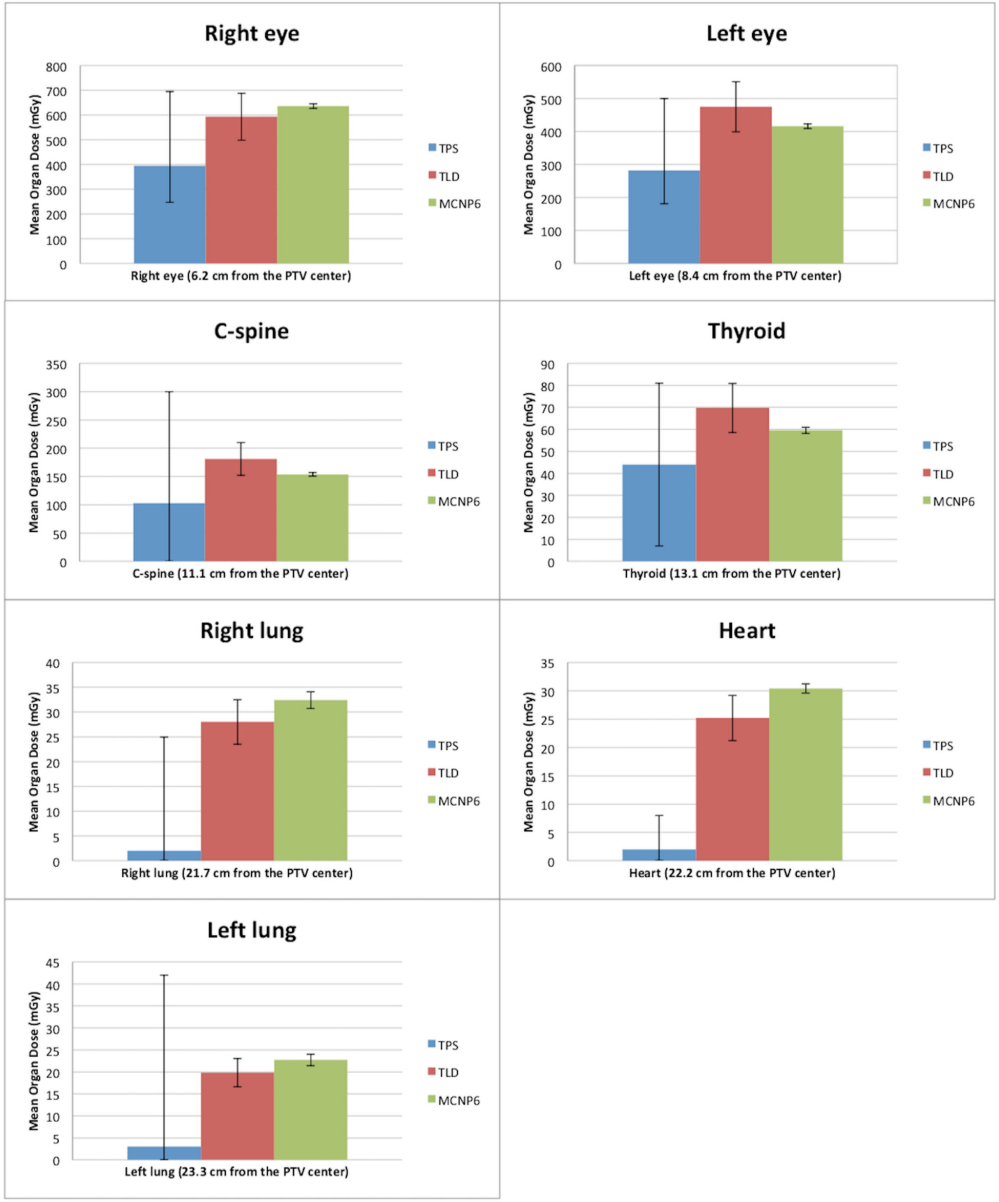
Comparison between doses outside the treatment fields obtained by TPS, TLDs, and MC for the 3DCRT technique. The error bars of the TPS dose calculations define the interval between the minimum and maximum calculated doses. The error bars of the measurements with the TLDs correspond to measurements of standard deviations. The error bars of the MC simulations correspond to the calculated uncertainty for each organ.

**Figure 3:**
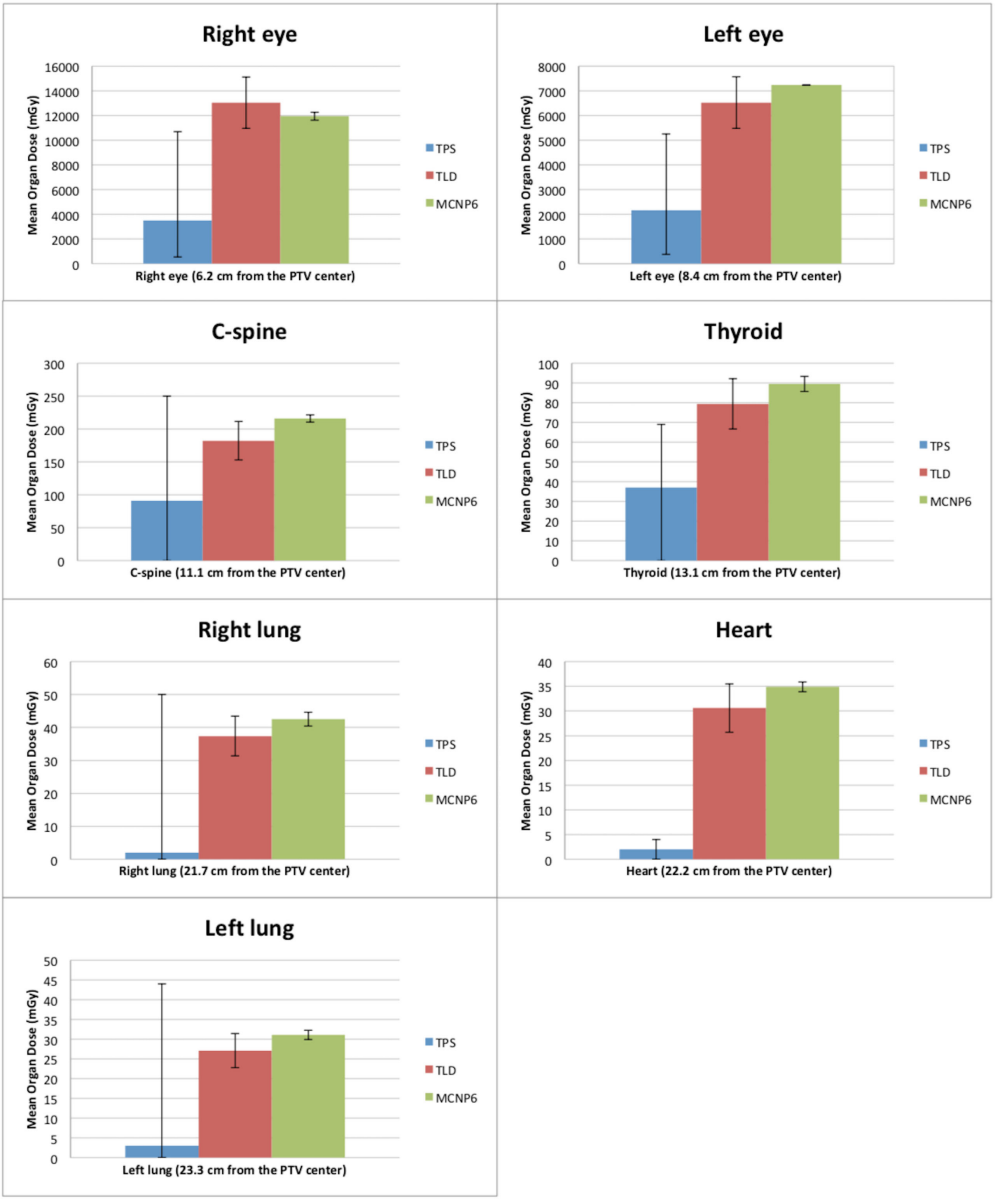
Comparison between doses outside the treatment fields obtained by TPS, TLDs, and MC for the IMRT technique. The error bars of the TPS dose calculations define the interval between the minimum and maximum calculated dose. The error bars of the measurements with the TLDs correspond to measurements of standard deviations. The error bars of the MC simulations correspond to the calculated uncertainty for each organ.

Measurements are considered the gold standard. The mean dose measured by TLDs for the 3DCRT and IMRT techniques is found in **Table 5**. **Figures 2**, **3** show the out-of-field doses in different OARs, obtained by the TPS calculations, the TLD measurements, and the MC simulations, for both techniques.

**Table 5:** Mean dose measured by TLDs in out-of-field organs.

Mean dose measured by TLDs (mGy) and corresponding standard deviation (SD, in mGy)				
Out-of-field organ	3DCRT	± SD	IMRT	± SD
Right eye (6.2 cm from PTV center)	593.0	93.7	13040.6	2060.4
Left eye (8.4 cm from PTV center)	475.2	75.1	6525.3	1031.0
C-spine (11.1 cm from PTV center)	180.9	28.6	182.3	28.8
Thyroid (13.1 cm from PTV center)	69.7	11.0	79.4	12.5
Right lung (21.7 cm from PTV center)	28.0	4.4	37.4	5.9
Heart (22.2 cm from PTV center)	25.2	4.0	30.6	4.8
Left lung (23.3 cm from PTV center)	19.8	3.1	27.1	4.3

The comparison between the dose calculations performed by the TPS and the doses measured with TLDs shows that the out-of-field dose values are always higher for the measurements with TLDs. In addition, the out-of-field measured doses with the IMRT technique are, on average, seven times higher than with the 3DCRT technique. **Figures 2**, **3** show that the difference between the dose calculation using the TPS and the dose measured by the TLDs increases in out-of-field organs at higher distances from the center of PTV.

Comparing the MC dose simulations with the TLD dose measurements (**Figures 2**, **3**), a better agreement (i.e., lower differences) between the out-of-field doses can be observed, with respect to the comparison between TPS calculations and TLD measurements. For both TLD measurements and MC simulations, there is an increase in dose, namely, dose is, on average, 6.5 times higher using the IMRT technique, when compared to the 3DCRT technique (**Figures 2**, **3**). For TPS calculations, there is also an increase in dose, namely, dose is, on average, 3.0 times higher using the IMRT technique, when compared to the 3DCRT technique.

**Figure 2** compares the out-of-field doses with the TPS calculations and the measured doses with the TLDs for the 3DCRT technique, showing that the TPS has lower dose values for all organs. The dose values with the TLDs are approximately 1.5, 1.7, 1.8, 1.6, 14, 13, and 6.7 times higher for the right eye, left eye, C-spine, thyroid, right lung, heart, and left lung, respectively, when compared to TPS calculations. **Figure 2** also relates the out-of-field doses with the MC and the measured doses with the TLDs for the 3DCRT technique. The dose values calculated with MC are about 1.1, 1.1, 1.2, and 1.2 times higher compared to TLD measurements, for the right eye, right lung, heart, and left lung, respectively. The doses measured with the TLDs are about 1.1, 1.2, and 1.2 times for the left eye, C-spine, and thyroid respectively, when compared to MC simulations.

**Figure 3** shows the out-of-field doses calculated with the TPS and the doses measured with the TLDs for the IMRT technique. The TPS yields lower dose values for all organs. The doses measured with the TLDs are approximately 3.7, 3.0, 2.0, 2.1, 18.5, 15.5, and 9.0 times higher for the right eye, left eye, C-spine, thyroid, right lung, heart, and left lung, respectively, when compared to TPS calculations. **Figure 3** also compares the out-of-field doses with simulations in MC and measured doses with TLDs for the IMRT technique. It is observed that doses are about 1.1, 1.2, 1.1, 1.2, 1.1, and 1.1 higher with MC simulations, compared to TLD measurements, for the left eye, C-spine, thyroid, right lung, heart, and left lung, respectively. For the right eye, a higher dose was found with the TLDs in about 1.1 times, when compared to MC simulations.

In short, for the 3DCRT technique, the average relative dose difference between MC simulations and TLD measurements is lower (14%) than the average relative dose difference between TPS-calculated dose values and TLD measurements (61%). For the IMRT technique, the average relative dose difference between MC simulations and TLD measurements is also lower (13%) than the average relative dose difference between TPS-calculated dose values and TLD measurements (74%). The agreement between TPS calculations and measurements is better for 3DCRT when compared with the IMRT technique, presumably due to a lower performance of the TPS calculation dose with the MLC movement in out-of-field regions.

Additionally, for organs at a distance up to 13 cm from the PTV, such as eyes, thyroid, and c-spine, a lower difference for MC vs. TLDs in comparison to a difference of TPS vs. TLDs was observed in all points. These dose differences are generally more pronounced for the organs further away (up to 23.3 cm) from the center of PTV, such as lungs and heart.

## Discussion

Although there are other publications on this topic, our study presents several innovative points that differentiate it from others, such as the following: (i) This work is based on an MC-detailed model of a LINAC head and specifically an MLC, previously validated, rather than relying on a built-in LINAC library present in other MC calculation programs, as in a large part of the studies presented in the literature. (ii) The movements of the different MLC leaves (in dynamic mode) were manually adjusted to each position of the treatment plan parameters in order to recreate a scenario closer to reality. (iii) The IMRT technique required a complex and new methodology to adjust all the parameters of the treatment plan to the computational pediatric phantom, namely, the different movements of the phantom in order to create the real movements of the treatment couch for each field. (iv) This study evaluates out-of-field doses with the IMRT technique by using measurements with TLDs, calculations with TPS, and calculations with MC methods for pediatric patients, and currently few studies in the literature combine these three approaches. In short, our study mimics an accurate and detailed MC model of a LINAC/MLC, a dose plan of a pediatric case using the IMRT technique with dynamic movement of the MLCs during irradiation, exhibiting a high degree of innovation and applied to a real scenario of clinical practice.

### TPS dose calculations

The literature pinpoints that commercial TPSs are not suitable for correctly assessing and characterizing the doses in out-of-field locations, which receive low doses, i.e., doses below 5% of the total delivery dose (7, 9, 45–47). The results of this study are in agreement with previous studies, in which the out-of-field doses calculated by the TPS are systematically underestimated, when compared with MC simulations and TLD measurements (46, 48–50). Huang *et al.* were the first to evaluate the accuracy of the out-of-field doses using the Pinnacle (3) TPS for the IMRT technique (7). Huang *et al.* found that the TPS calculation significantly underestimated the out-of-field doses for both dynamic IMRT and step-and-shoot IMRT (7). In step-and-shoot IMRT, they obtained an underestimation by an average of 50%. Nonetheless, we should bear in mind the different dynamic IMRT treatment, TPS, and calculation algorithm used.

Our results report that the doses calculated by the TPS in the organs more distant from PTV (lungs and heart) are approximately the same for both 3DCRT and IMRT. Furthermore, in out-of-field organs closest to the PTV center, such as the eyes, the doses calculated by the TPS show higher values for the IMRT, when compared to the 3DCRT technique.

The lack of accuracy of the TPS calculation in organs outside and farther away from the treatment fields may be related to the dose discrepancies reported by TPS, which is probably due to the simplification of the AAA algorithm, which makes it faster and feasible in routine radiotherapy treatments. The dose in the eyes is higher, considering that these structures are closer to the field edge, when comparing to organs further away from PTV, such as the lungs and the heart. Howel et al. (46) also reported that as the distance to the field boundary increases, the underestimation of the dose by the TPS also increases.

### TLD measurements

Roger Harrison (51) points out that, in general, the out-of-field doses in radiotherapy vary in an extended range, between 10.0 and 60.0 Gy. For the 3DCRT technique, our study yielded out-of-field doses from 19.8 mGy to 593.0 mGy for measurements with TLDs. For the IMRT technique, out-of-field doses ranged from 27.0 to 13,041.0 mGy, for a total prescribed dose of 54.0 Gy in both techniques. These results should be considered with particular attention, as these are doses that could be delivered to pediatric patients and may increase the risk of development of a secondary cancer. The out-of-field measurements obtained in this study have a relative uncertainty of around 16% (k = 1) and were performed using an anthropomorphic physical pediatric phantom using TLDs. The work performed by Knežević *et al.* (32), reports a TLD uncertainty of ≈2.9% for doses below 2 mGy and ≈4.2% for doses below 2 mGy. This difference could be explained by the different approaches related to the calibration of the TLDs between the two studies, namely, in the reader calibration factor.

### MC simulations

The MC simulations were extremely demanding, as well as innovative, since the movements of the MLCs were manually modified in the MLC/LINAC model script. Given the dynamic movement of the MLC, about 140 simulations of different leaf positions were performed. Therefore, part of the innovation of this study is related to the differentiation of manual adjustments in the beam geometry in order to represent more realistically and accurately all the geometry concerning the MLC movement that is required by the IMRT technique.

In this study, differences between dose simulation with MC and measurements with TLDs were obtained at up to 21.2% and 18.5% for the 3DCRT and IMRT techniques, respectively. In a recent study by Sánchez-Nieto et al. (52), dose differences outside treatment fields of up to about 20% were obtained between MC simulations and measurements with ionization chambers. These differences are also similar to those reported by Joosten et al. (47), Kry et al. (53), and Bednarz and Xu (34). On the one hand, in our study the differences between the 3DCRT and IMRT techniques could be explained by the MLC positioning approximations in order to mimic the dynamic IMRT treatment. In other words, in our MC model it was not possible to recreate a full IMRT treatment, as was possible with the 3DCRT treatment, due to the difficulty of not being able to move the leaves during the simulated irradiation. On the other hand, we have obtained discrepancies between measurements and MC simulation between 13% and 14% that could be explained by the 15.8% uncertainty of the TLD measurements.

### Comparison between TLD measurements, TPS, and MC simulations

The results of this study report the novel comparison between measurements and calculations in TPS and simulations of out-of-field doses, in a detailed MC model, mimicking the IMRT dose distributions in a computational phantom. The out-of-field doses for the IMRT technique were calculated for the first time, using MCNP6 and the pediatric computational voxel phantom combined with the Varian Clinac 2100 CD model, and also measured using TLDs placed in the pediatric physical phantom. For both TLD measurements and MC simulations, the IMRT technique yielded higher doses with respect to the IMRT technique. Although it is well known that TPSs are not commissioned to evaluate the out-of-field doses and our work verified an underestimation of these doses, an increasing underestimation with increasing distance from the treatment field edge was also verified. In our study, the organs located nearest to the PTV show lower relative differences with respect to TLD measurements or MC simulations, compared to organs located further away from the PTV. In organs nearest to the PTV such as the eyes, the TPS calculated the dose with the IMRT technique which is an order of magnitude higher than with the 3DCRT technique.

Some studies available in the literature report on measurements of out-of-field doses and compared them with doses calculated by TPS. Other researchers also used MC simulations to evaluate the out-of-field doses provided by the TPS software (2, 9). The findings of these studies vary and depend on the treatment modality and on the anatomical location of the target volumes (9). Joosten *et al.* found that in the first 10 cm outside the treatment fields, MC dose simulations are more accurate than those the dose calculations of commercial TPS (47). Our work reports that up to approximately 23 cm from the center of the target volume, the MC-based dose simulations produce more accurate results than the doses calculated by commercially available TPSs. The highest differences between phantom measurements and dose calculations were 20% for MC simulations and 179% for TPS calculations (47). Although our results report a large discrepancy between the dose calculation with the TPS and the dose measurement with the TLD, small discrepancies are found between dose simulation with MC and dose measurement with the TLDs.

Furthermore, in contrast to our results, Majer *et al.* show higher doses in the C-spine and thyroid for the 3DCRT technique when compared to the IMRT technique, both for measurements with TLDs and for dose calculations with TPS (54). We suggest that the differences between the results obtained in this study and the results obtained by Majer *et al.* are due to the following. i) Differences in PTV locations and volumes: in Majer *et al.*’s (54) study, the PTV was spherical and located in the left-anterior side, and in our study the PTV was elliptical and located in the right-frontal side of the brain. ii) Different treatment plans: Majer et al. (54) performed an IMRT plan with nine coplanar fields and for 3DCRT three non-coplanar fields. We have performed an IMRT plan with seven coplanar fields and six non-coplanar fields for the 3DCRT technique. (iii) Different versions of the TPS: Majer *et al.* (54) performed the dose plans with Eclipse TPS version 8.6, and our study was performed with 13.0 version of the same TPS.

Beierholm *et al.* (48) conducted a study in which they measured the out-of-field doses in brain tumors in a pediatric phantom with TLDs, considering a prescribed dose of 54 Gy in 30 fractions. Beierholm *et al.* obtained for the thyroid a dose of 103.4 mGy with VMAT for a PTV smaller than 2 cm. Our results show that the thyroid was exposed to 69.7 and 79 mGy with 3DCRT and with IMRT, respectively. Although our work does not evaluate dose plans with VMAT, the MC model developed could be applied to VMAT treatments.

According to the literature, the out-of-field doses are higher using IMRT (8, 55), when compared to the 3DCRT technique. The doses obtained from our study support the literature because the doses for 3DCRT ranged from 20 to 590 mGy and the doses for IMRT ranged from 27 to 13,040 mGy.

According to Paganetti (11), about 50% of all second tumors seem to develop with doses delivered in tissues receiving less than 2,500 mGy (11). For the right eye, the obtained results by measurements of our study show that the IMRT technique yields doses about 13,040 mGy, for the left eye doses about 6,525 mGy, corroborating the concern about the out-of-field doses. These results may raise some concerns, as the dose tolerance tables for OARs only mention that the eyes should have a Dmax below 45–50 Gy (24, 25). Although our phantom has no eye lens, the findings of a recent study (56) showed a significantly higher cataract incidence in eyes that had received a maximum dose of 5 Gy in the eye lens. Additionally, the dose tolerance tables are created on the basis of retrospective results, always considering that the aim of radiotherapy treatment is to deliver the highest dose to the PTV and the lowest dose in the OARs. Tolerance doses in radiotherapy are not defined with special attention to the effects of low doses in the different organs and the individual risk analysis of developing secondary tumors after irradiation. Moreover, the authors of this study consider that for radiotherapy planning, there are organs/tissues further away and also close from the field edge that should be outlined, as OARs in order to consider possible side effects, especially relevant in pediatric patients.

In the present study, a specific and simplified clinical case of a tumor volume, defined in a phantom, was analyzed. However, the results in this study can be used by clinicians for better understanding of the possible risks that out-of-field doses carry, especially in pediatric patients.

### Pediatric radiotherapy, second cancers—the way forward

In conclusion, despite the limitations of this study, namely, the simulation of a single scenario/tumor volume, the obtained results seem to indicate that out-of-field doses are higher with IMRT, compared to 3DCRT.

The obtained results indicate that out-of-field organs and tissue doses assessed in this work are of concern as they may presumably increase the risk of development of second tumors. It must, however, be emphasized that the decision on the type of radiotherapy treatment and modality should always be taken by the radiation oncologists, considering all the clinically relevant information about the patient.

Prospectively, on the basis of the methodology used, as well as the results obtained in this and other future studies, it will be possible to create an out-of-field dose database, which may have information from adult and pediatric patients. Such a database may improve radiotherapy treatment planning, as it allows the identification of dosimetric characteristics that may lead to higher out-of-field doses.

The methodology followed in this study, namely, the MC simulations combined with a Varian LINAC and pediatric phantom computational models, will allow the development of applications and tools that may benefit from individualized information, such as individualized dose assessment methodologies, retrospective dosimetric calculations for epidemiological studies, and radiological risk estimation models, to estimate the development of secondary cancers.

## Summary

As widely described in the literature, dose measurements in a clinical environment lead to more realistic and detailed data on organ doses (54) in radiotherapy treatments and allow validation and benchmarking of doses calculated by TPS and simulated with MC methods. The results obtained in this study contribute to a better understanding of doses outside of treatment fields in pediatric patients, and these types of studies are scarce in the literature. Additionally, the present work combines, in a unique way, MC simulations, TPS calculations, and TLD measurements, using a pediatric voxel phantom segmented from CT images, the original pediatric physical phantom and a developed model of the LINAC, which includes the MLC when IMRT is considered. Although in radiotherapy planning, doses outside the treatment fields in the order of cGy up to 2–3 Gy are often neglected, from the point of view of radiological protection and radiosensitivity, these doses cannot be ignored (2), especially for pediatric patients, because they can cause radiation-induced tumors in healthy organs and tissues.

## Data availability statement

The datasets presented in this article are not readily available because we have a confidentiality agreement with Varian Medical Systems. Requests to access the datasets should be directed to AS, anacravosa@ctn.tecnico.ulisboa.pt.

## Author contributions

AS, PA, and PV contributed to conception and design of the study. AS, AB, and TM performed the treatment plans and phantom irradiation. AS and BB perform the LINAC Monte Carlo model. AS wrote the first draft of the manuscript. PA, PV, AB, TM, and BB wrote sections of the manuscript. All authors contributed to manuscript revision, read, and approved the submitted version

## Funding

This research was partially funded by Fundação para a Ciência e Tecnologia (FCT)/Portugal through the contract UIDB/04349/2020).

## Conflict of interest

The authors declare that the research was conducted in the absence of any commercial or financial relationships that could be construed as a potential conflict of interest.

## Publisher’s note

All claims expressed in this article are solely those of the authors and do not necessarily represent those of their affiliated organizations, or those of the publisher, the editors and the reviewers. Any product that may be evaluated in this article, or claim that may be made by its manufacturer, is not guaranteed or endorsed by the publisher.
