# Corrigendum: Comparison of 3DCRT and IMRT out-of-field doses in pediatric patients using Monte Carlo simulations with treatment planning system calculations and measurements

**DOI:** 10.3389/fonc.2023.1293922

**Published:** 2023-10-09

**Authors:** Ana Cravo Sá, Andreia Barateiro, Bryan P. Bednarz, Pedro Almeida, Pedro Vaz, Tiago Madaleno

**Affiliations:** ^1^ Radiation Protection and Safety Group, Centro de Ciências e Tecnologias Nucleares (C2TN), Bobadela, Portugal; ^2^ Diagnostic, Therapeutic and Public Health Sciences Department, Escola Superior de Tecnologia da Saúde de Lisboa (ESTeSL), Lisbon, Portugal; ^3^ Instituto de Biofísica e Engenharia Biomédica, Faculdade de Ciências, Universidade de Lisboa, Lisbon, Portugal; ^4^ Radiotherapy Department, Portuguese Institute of Oncology Francisco Gentil, Lisbon, Portugal; ^5^ Department of Medical Physics, Wisconsin Institutes for Medical Research, University of Wisconsin Hospital and Clinics, Madison, WI, United States

**Keywords:** radiotherapy planning, out-of-field dose, pediatric tumors, Monte Carlo simulations, computational voxel phantoms, IMRT, 3DCRT

In the published article, there was an error in the Funding statement. The authors mistakenly mentioned that there was no funding. The correct Funding statement appears below.

Funding

This research was partially funded by Fundação para a Ciência e Tecnologia (FCT)/Portugal through the contract UID/Multi/04349/2019).

The authors apologize for this error and state that this does not change the scientific conclusions of the article in any way. The original article has been updated.

